# Distinct maternal metabolites are associated with obesity and glucose-insulin axis in the first trimester of pregnancy

**DOI:** 10.1038/s41366-023-01295-4

**Published:** 2023-04-07

**Authors:** Julia Bandres-Meriz, Christina Kunz, Jesper F. Havelund, Nils J. Færgeman, Alejandro Majali-Martinez, Regina Ensenauer, Gernot Desoye

**Affiliations:** 1 Department of Obstetrics and Gynaecology, Medical University of Graz, Graz, Austria; 2 Department of Biochemistry and Molecular Biology, University of Southern Denmark, Odense M, Denmark; 3 Institute of Child Nutrition, Max Rubner-Institut, Federal Research Institute of Nutrition and Food, Karlsruhe, Germany

**Keywords:** Metabolic syndrome, Biochemistry

## Abstract

**Background/Objectives:**

Obesity in pregnancy associates with changes in the glucose-insulin axis. We hypothesized that these changes affect the maternal metabolome already in the first trimester of human pregnancy and, thus, aimed to identify these metabolites.

**Patients/Methods:**

We performed untargeted metabolomics (HPLC-MS/MS) on maternal serum (*n* = 181, gestational weeks 4^+0^–11^+6^). For further analysis, we included only non-smoking women as assessed by serum cotinine levels (ELISA) (*n* = 111). In addition to body mass index (BMI) and leptin as measures of obesity and adiposity, we metabolically phenotyped women by their fasting glucose, C-peptide and insulin sensitivity (IS_HOMA_ index). To identify metabolites (outcome) associated with BMI, leptin, glucose, C-peptide and/or IS_HOMA_ (exposures), we used a combination of univariable and multivariable regression analyses with multiple confounders and machine learning methods (Partial Least Squares Discriminant Analysis, Random Forest and Support Vector Machine). Additional statistical tests confirmed robustness of results. Furthermore, we performed network analyses (MoDentify package) to identify sets of correlating metabolites that are coordinately regulated by the exposures.

**Results:**

We detected 2449 serum features of which 277 were annotated. After stringent analysis, 15 metabolites associated with at least one exposure (BMI, leptin, glucose, C-peptide, IS_HOMA_). Among these, palmitoleoyl ethanolamine (POEA), an endocannabinoid-like lipid endogenously synthesized from palmitoleic acid, and N-acetyl-L-alanine were consistently associated with C-peptide in all the analyses (95% CI: 0.10–0.34; effect size: 21%; *p* < 0.001; 95% CI: 0.04–0.10; effect size: 7%; *p* < 0.001). In network analysis, most features correlating with palmitoleoyl ethanolamide and N-acetyl-L-alanine and associated with C-peptide, were amino acids or dipeptides (*n* = 9, 35%), followed by lipids (*n* = 7, 27%).

**Conclusions:**

We conclude that the metabolome of pregnant women with overweight/obesity is already altered early in pregnancy because of associated changes of C-peptide. Changes of palmitoleoyl ethanolamide concentration in pregnant women with obesity-associated hyperinsulinemia may reflect dysfunctional endocannabinoid-like signalling.

## Introduction

The number of women with obesity of reproductive age has increased in the last decades contributing to a variety of pregnancy complications including gestational diabetes mellitus (GDM) and large for gestational age newborns [1–4]. Also, offspring born to mothers with obesity have increased risk of developing obesity in adulthood [5–7].

The relationship of the maternal metabolome in the second half of pregnancy with pregnancy outcomes has been well characterized [8–16]. However, there is little information about the maternal metabolome and its changes in early pregnancy in conditions with disturbed metabolism, such as obesity, despite evidence of early metabolic changes associating with adverse pregnancy outcomes [17]. In a targeted metabolomics analysis early in the second trimester, some fatty acids and lipids were positively associated with body mass index (BMI) [12]. Recently, we demonstrated that the metabolism of pregnant women with overweight and obesity is altered already very early in pregnancy, i.e., from 4 weeks onward. In particular, overweight and obesity were associated with increased C-peptide concentration and reduced insulin sensitivity, but unchanged glucose concentrations, compared to normal weight pregnant women [18]. Also, fatty acids were changed, not by BMI, but rather by C-peptide and insulin sensitivity (IS_HOMA_), which were associated with decreased concentrations of omega-3 polyunsaturated fatty acids [19].

A thorough characterization of the maternal metabolome already early in pregnancy is clinically relevant, because this time period may offer an intervention window for medical and dietary counselling to reduce the risk of adverse pregnancy outcomes.

In the present study, we aimed to identify changes in the maternal serum metabolome in the obesity-associated environment of the first trimester of pregnancy. Obesity, as a BMI-based categorization, is often associated with complex metabolic alterations. However, individuals with obesity can also be metabolically ‘healthy’ [20]. In a previous study using targeted metabolomics, maternal metabolites correlated with BMI, glucose and insulin sensitivity in the second half of pregnancy [15]. Hence, to address changes in the maternal metabolome, other exposure variables, in addition to BMI, need consideration.

In this study, we used untargeted metabolomics to analyse the association of maternal obesity and adiposity (BMI and leptin, respectively) with maternal serum metabolites (outcome). To account for potential obesity heterogeneity, we also included parameters of the glucose-insulin axis (glucose, C-peptide and IS_HOMA_).

## Materials, participants and methods

### Participants

The study was conducted in a non-academic setting between May 2017 and August 2018. Women with known co-morbidities, i.e., pre-existing diabetes mellitus, hypertension and auto-immune diseases, were excluded from the study. A total of 181 pregnant women (age ≥ 18 years) were recruited at the moment of voluntary pregnancy termination for psychosocial reasons (gestational age 4^+0^–11^+6^ weeks based on the last menstrual period). Smokers were excluded, resulting in 111 pregnant women included in the study. The study was approved by the ethics committee of the Medical University of Graz no. 29-095 ex 16/17, 23rd December 2016 and 31-094 ex 18/19, 1 March 2019.

This is a basic science study which aims at generating hypotheses for further research. The exploratory design of the study and the use of untargeted metabolomics precludes calculating sample size.

### Sample collection and analyses

Venous blood (8 ml) was collected after overnight fasting. The serum fraction was separated by centrifugation (2000 × *g* at 4 °C for 10 min) and immediately stored at −80 °C until further use, as previously described [18].

Smoking status was assessed in a two-step process. First, participants self-reported their smoking habits (yes/no) in a questionnaire. Second, of all participants that self-reported as non-smokers, serum cotinine concentration was quantified with a competitive immunoassay as described [18]. Only participants that reported to be non-smokers and whose cotinine concentration in serum was under the detection limit were included in the study. Maternal weight and height were measured at the day of pregnancy termination. BMI [kg/m^2^] was calculated as weight at the time of pregnancy termination [kg] divided by the square of height [m]. Serum leptin concentration [ng/ml] was measured by a sandwich immunoassay (DRG, Marburg, Germany, Cat# EIA2395) and used as a proxy for maternal fat mass.

Fasting serum glucose [mg/dl] was measured using the hexokinase-based test (Glucose HK Gen.3, Roche Diagnostics, Mannheim, Germany) on an automated analyzer (Cobas® 8000 c701, Roche Diagnostics, Mannheim, Germany). Fasting serum C-peptide [pmol/l] was measured by a Sandwich Immunoassay (R&D Systems, Minneapolis, MINN, USA Cat# DICP00). Fasting serum glucose and fasting C-peptide concentrations were used to calculate the homeostatic model assessment of insulin sensitivity (IS_HOMA_) [21].

### Metabolomics analysis and data processing

Metabolites were extracted from 100 µl of serum following a Folch´s like extraction procedure. In brief, the serum samples were diluted in 600 µl of a chloroform/methanol (2:1; v/v) solution and incubated in a thermoshaker at 1000 rpm, 4 °C for 1 h. Thereafter, 200 µl of water were added per sample and incubated at 1000 rpm, 4 °C for 15 min. This was followed by two consecutive centrifugation steps at 13,000 rpm, 4 °C for 15 min. The aqueous phase was diluted in 200 µl of a chloroform/methanol/water (86/14/1; v/v/v) solution, followed by incubation at 1000 rpm, 4 °C for 15 min. The aqueous phase was lyophilized and resuspended in 30 µl 1% formic acid. For metabolomic analysis, 2 µl were injected using a Vanquish Horizon HPLC (Thermo Fisher Scientific, Germering, Germany) coupled to a Q Exactive HF mass spectrometer (Thermo Fisher Scientific, Bremen, Germany) for mass spectrometric analysis (for further information see Supplementary Materials 1).

### Statistical analysis

Maternal characteristics (maternal age, gestational age, leptin, glucose, C-peptide and IS_HOMA_) were compared between the BMI groups (underweight-normal weight, overweight-obese) by Kruskal–Wallis tests.

**Fig. 1:**
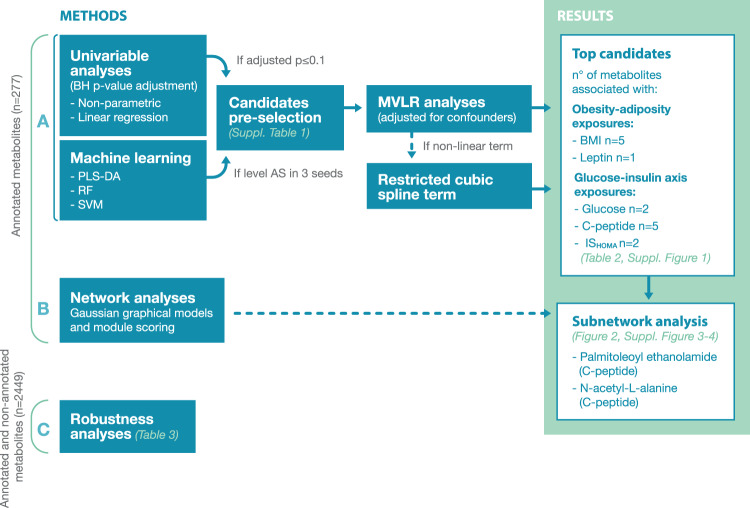
Flow chart of data analyses. **A** Univariable analysis and machine learning methods were combined to pre-select metabolic candidates (Supplementary Table 1). To select the most prominent candidates, multivariable linear regression (adjusting for confounders)—with restricted cubic spline terms if association was non-linear—were performed (Table 2 and Supplementary Fig. 1). **B** Network analysis was applied to identify sets of correlating metabolites that are coordinately regulated by an exposure (BMI, leptin, glucose, C-peptide or IS_HOMA_). Subnetworks comprising metabolites of interest, i.e palmitoleoyl ethanolamide and N-acetyl-L-alanine, were further explored (Fig. 2, Supplementary Figs. 3, 4). **C** To examine the robustness of the results, univariable analyses were performed in the total set of metabolites (annotated and non-annotated) (Table 3). BH Benjamini-Hochberg, PLS-DA partial least squares discriminant analysis, RF random forest, SVM support vector machine, MVLR multivariable linear regression analysis, BMI body mass index, IS_HOMA_ homeostatic model assessment of insulin sensitivity.

The flow chart of data analyses to identify significant metabolites is summarized in Fig. 1. Analyses were performed with glucose concentrations in mg/dl, which were converted into mmol/l for reporting cohort characteristics. All statistical analyses were performed with R (version 4.0.4) [22] and MetaboAnalyst (version 5.0) [23]. Log2-transformed values of the metabolites were used for all further analyses (for further pre-processing see Supplementary Materials 2.1).

#### Univariable analysis

The following variables were analyzed as exposures: maternal BMI (continuous and categorized as underweight-normal weight or overweight-obese) and leptin concentration, glucose concentration, C-peptide and IS_HOMA_ index (all continuous and categorized by below/above median). Associations between metabolites abundances and exposure variables were analyzed with univariable analyses (Supplementary Materials 2.2). *P*-values were adjusted for multiple testing by the method of Benjamini-Hochberg [24], which allows controlling for the false discovery rate (FDR).

#### Machine learning analysis

In addition, the total set of annotated metabolites was analyzed for its ability to classify the binarized exposures (categorized by their median) using the R-package “biosigner” [24]. The package implements a wrapper for the binary classifiers partial least squares discriminant analysis (PLS-DA), random forest (RF) and support vector machine (SVM) (Supplementary Materials 2.3). For each binarized exposure, metabolites were selected with “biosigner” three consecutive times, starting with different random seeds.

For further analyses, i.e. multivariable analysis adjusted for confounders, metabolites which fulfilled at least one of the two criteria: (1) the adjusted *p*-value is ≤0.1 in univariable analyses, (2) the metabolite is selected by “biosigner” for all three random seeds were selected.

#### Multivariable regression analysis

Identified metabolites were further characterized by multivariable regression models. These included one of the five continuous exposures and the following confounders: gestational age [days], maternal age [years], processing time of blood [minutes] and the interaction between the respective continuous exposure and gestational age. In a first step, linear regression models were used for multivariable analyses. Linearity of the model terms for each influencing variable was then tested by Tukey’s test for a quadratic form of the model term. If Tukey’s test showed a significant violation of the linear assumption, i.e., *p*-value ≤ 0.05, we replaced the linear term of this influence variable by a restricted cubic spline with three knots (at the 10%, 50%, and 90% quantile of the influence variable) in further multivariable regression analysis.

Analysis of variance of the regression models was performed and *p*-value of *F*-tests are reported as well as effect estimates of the exposure together with 95% confidence intervals, which are based on characteristic effects, i.e., changes, in the exposures. The effect of the exposure is the change in the metabolite measurements when inserting in the model the upper bound of the characteristic effect compared to inserting the lower bound of the characteristic effect of the exposure, while holding the interacting confounder gestational age fixed at its median value within the cohort.

Because of the exploratory design of the study, the results of the multivariable analyses were not adjusted for multiple testing.

#### Robustness analysis

To determine the robustness of the results, univariable analyses including all the measured metabolites (*n* = 2449, annotated and non-annotated) were performed. A metabolite was considered significantly associated with the exposure if the adjusted *p*-value was ≤0.1 (cf. “Univariable analysis” above).

#### Network analysis

Phenotype-driven metabolomics network analysis was performed as an additional statistical approach to screen for further metabolites. The method of Do et al. implemented in R-package “MoDentify” [25], was used for exposure-driven module identification in the metabolomics network (for further details see Supplementary Materials 2.4). Metabolomic networks were visualized in Cytoscape (version 3.8.2) [26].

#### Subnetwork and pathway analysis

For subnetwork and pathway enrichment analysis, metabolites that fulfilled the following criteria: (1) significantly associated with an exposure in the multivariable analysis adjusted for confounders, and (2) the association remained significant in the robustness analysis were selected. For metabolites meeting both criteria, subnetworks were constructed (out of the MoDentify network) including this metabolite. Among these subnetworks, those with the highest number of metabolites were selected for graphical representation in Cytoscape and pathway analysis (MetaboAnalyst 5.0) [27, 28]. All metabolites encompassed in the subnetwork were selected for pathway enrichment analysis. The human metabolome data base (HMDB) identifiers were used for enrichment analysis in MetaboAnalyst and compared to the KEGG library for identification of human metabolic pathways [29], and the libraries PubChem [30] and ChEBI [31] for chemical structures for identification of metabolite chemical species. Only metabolites containing at least two entries in the KEGG library were considered for pathway enrichment. The globaltest algorithm [27] was used for overrepresentation enrichment analysis and only enrichments that deemed significant after Holm p-value adjustment (FDR ≤ 0.05) were considered for graphical representation.

#### Sub-cohort analysis—healthy and unhealthy obesity

To investigate differences in the metabolome of women with healthy and unhealthy obesity, we classified the women based on their insulin sensitivity using the IQR IS_HOMA_ of the total cohort (*n* = 111, IQR 0.5–1.0) for classification. Women whose IS_HOMA_ < 0.5 were classified as low insulin sensitive and women whose IS_HOMA_ ≥ 0.5 as normal insulin sensitive. IS_HOMA_ information was missing for two samples, which were, therefore, excluded from the subanalysis.

Metabolites (*n* = 19) identified in the pre-selection step (Supplementary Table 1) were compared between the low and normal insulin sensitive groups by Kruskal–Wallis test and linear regression. *P*-values were adjusted by the method of Benjamini-Hochberg and the association was considered significant if the adjusted *p*-value ≤ 0.1. Metabolites that were significantly different between groups were further characterized by multivariable linear regression analysis. These included IS_HOMA_ as exposure and the following confounders: gestational age [days], maternal age [years], processing time of blood [min].

## Results

### Study participants

The study included 111 pregnant women (maternal age [median, IQR]: 31.5, 26.0–38.0; gestational age [median, IQR]: 7^+0^, 7^+5^ – 8^+5^ weeks based on the last menstrual period). Of these, 77 were underweight-normal weight (BMI < 25 kg/m^2^, 69.4%), 28 had overweight (25 ≤ BMI < 30 kg/m^2^, 25.2%) and six had obesity (BMI ≥ 30 kg/m^2^, 5.4%). The groups with overweight and obesity were combined (BMI ≥ 25 kg/m^2^, *n* = 34, 30.6%) and maternal characteristics compared between the underweight-normal weight (BMI < 25 kg/m^2^) and the group with overweight-obesity (BMI ≥ *25* kg/m^2^) (Table 1). Leptin (*p* < 0.001) and C-peptide (*p* = 0.009) concentrations were significantly increased, and insulin sensitivity decreased (*p* = 0.002) in the group with overweight-obesity compared to the underweight-normal weight group. No significant differences in fasting glucose were found between both groups.

**Table 1:** Cohort characteristics stratified by maternal BMI.

	BMI < 25 kg/m^2^			BMI ≥ 25 kg/m^*2*^			
	(*n* = 77)			(*n* = 34)			
	*n*	median	IQR	*n*	median	IQR	*p*-value
Gestational age (days)	77	50	42–63	34	42	35–50	0.03
Maternal age (years)	76	31	25–38	34	34	27–39	0.23
Leptin (ng/ml)	77	9.4	5.8–14.5	34	16.1	11.6–24.2	<0.001
Glucose (mmol/l)	74	4.6	4.1–5.1	32	4.8	4.2–5.4	0.22
C-peptide (pmol/l)	77	355.1	269.8–435.5	34	437.3	332.2–523.3	0.009
IS_HOMA_ index	73	0.8	0.6–1.0	32	0.6	0.5–0.8	0.002

*BMI* body mass index (BMI < 25 kg/m^2^: underweight-normal weight; BMI ≥ 25 kg/m^2^: overweight-obese), *IS*_*HOMA*_ homeostatic model assessment of insulin sensitivity, *IQR* interquartile range.

Given are the *p*-values of the Kruskal–Wallis test comparing BMI groups.

### Selection of metabolite candidates

To reduce the number of variables and to enable biological interpretation of the results, only metabolites with known chemical structure (annotated metabolites, *n* = 277) were selected for statistical analysis. A total of 24 annotated metabolites were significantly associated with at least one exposure (BMI, leptin, glucose, C-peptide and IS_HOMA_) at the pre-selection step (Supplementary Table 1). We identified 15 metabolites significantly associated with the exposures (Table 2, Supplementary Fig. 1). Among these, six metabolites were associated with measures of obesity and adiposity (BMI and leptin) and nine metabolites with parameters of the glucose-insulin axis (glucose, C-peptide, IS_HOMA_).

**Table 2:** Metabolites significantly associated with BMI, leptin, glucose, C-peptide and IS_HOMA_.

Metabolite	Exposure	Effect estimate	Confidence interval (95%)	Effect Size (%)	*p*-value
*Obesity/Adiposity*					
Serine-Tyrosine	BMI	0.22	0.12 to 0.31*	0.30	<0.001
Phenylalanine-Threonine		0.22	0.14 to 0.31*	0.27	<0.001
Stachydrine		−0.14	−0.22 to −0.06	0.18	<0.001
Proline-Hydroxyproline		−0.04	−0.07 to −0.02	0.23	0.007
S-methyl-L-cysteine		−0.06	−0.09 to −0.02	0.12	0.002
Phenylalanine-valine	Leptin	0.16	0.04 to 0.29	0.17	0.021
*Glucose-insulin axis*					
Hexamethylphosphoramide	Glucose	0.20	−0.02 to 0.41	0.17	0.034
15S-hydroperoxy-11Z,13E-eicosadienoic acid		−0.12	−0.21 to −0.30	0.17	0.019
Palmitoleoyl ethanolamide	C-peptide	0.22	0.10 to 0.34	0.21	<0.001
Androsterone glucuronide		0.36	0.12 to 0.60*	0.21	0.050
N-acetyl-L-alanine		0.07	0.04 to 0.10	0.18	<0.001
7α,17α-dimethyl-5β-androstane-3α,17β-diol glucuronide		−0.03	−0.20 to 0.15*	0.17	0.003
Uridine		−0.05	−0.14 to 0.04*	0.13	0.004
S-Methyl-L-cysteine	IS_HOMA_	0.01	−0.02 to 0.04	0.11	0.004
1-Myristoyl-sn-glycero-3-phosphocholine		−0.07	−0.11 to −0.02	0.21	0.001

The influence of the exposures on the metabolites is modelled using linear regression terms or restricted cubic spline terms, as appropriate. The model is adjusted, assuming linear relationships, for gestational age [days], maternal age [years] and processing time [minutes] and includes an interaction term between the exposure and gestational age. In the multivariable models, the effect of the exposure within the interaction term was estimated by keeping gestational age fixed at its median value within the cohort (median gestational age [days] = 49). The effect estimates are given in relation to changes, i.e. characteristic effects, in the exposures: maternal BMI [kg/m^2^] = 20–21, C-peptide [pmol/l] = 100–200, leptin [ng/ml] = 10–20, glucose [mg/dl] = 10–20, IS_HOMA_ = 0.1–0.2. The 95% confidence intervals are given for the effect estimates. Effect size (%) refers to the coefficient of determination (R2) and is the proportion of the variance in the outcome variable that is predictable from the influence variables (R2 lies between 0 and 100%). Analyses of variance of the regression models were performed and *p*-values of *F*-tests are reported. * The association was not linear and, therefore, modelled with restricted cubic spline terms, see Supplementary Fig. 1 for information on shapes of selected spline term.

Maternal BMI was associated with five metabolites, all of which were amino acids (stachydrine and S-methyl-L-cysteine) or dipeptides (serine-tyrosine, phenylalanine-threonine, proline-hydroxyproline), whereas maternal leptin was only associated with one dipeptide (phenylalanine-valine).

Maternal glucose was associated with a modified fatty acid (15S-hydroperoxy-11Z,13E-eicosadienoic acid) and a phosphoramide (hexamethylphosphoramide). Maternal C-peptide was associated with five metabolites, comprising one lipid (palmitoleoyl ethanolamide), two sex steroid hormones (androsterone glucuronide and 7α, 17α-dimethyl-5β-androstane-3α, 17β.-diol glucuronide), one amino acid (N-acetyl-L-alanine) and one nucleoside (uridine). Maternal IS_HOMA_ was associated with one phospholipid (1-myristoyl-sn-glycero-3-phosphocholine) and one amino acid (S-methyl-L-cysteine).

### Robustness of the results

To assess the robustness of our findings, we additionally analysed all detected metabolites, both annotated and non-annotated (*n* = 2449). One-hundred and six metabolites (4.3% of the total number of metabolites) were associated with at least one of the exposures (number of metabolites: BMI = 4, leptin = 1, glucose = 67, C-peptide = 33, IS_HOMA_ = 1) (Supplementary Fig. 2). Among these, only eight metabolites had a known chemical structure (annotated) (Table 3). Interestingly, palmitoleoyl ethanolamide and N-acetyl-L-alanine were again significantly associated with C-peptide, making them robust candidates for further analyses.

**Table 3:** Robustness analysis.

		Continuous		Categorized	
Metabolite	Exposure	non-parametric (adj. *p*-value)	linear regression (adj. *p*-value)	non-parametric (adj. *p*-value)	linear regression (adj. *p*-value)
NA	BMI				
NA	Leptin				
Serine-Tyrosine	Glucose			*	
(S)-Malate				*	*
3,5-Dihydroxyphenyl dodecyl benzene-1,3-diol		*	*	*	
S-Methyl-L-cysteine		*		*	
N-acetyl-L-alanine	C-peptide		*		
Palmitoleoyl ethanolamide			*		
3-Hydroxybutyrylcarnitine		*	*		
γ-Glutamylleucine			*		
NA	IS_HOMA_				

Annotated metabolites within the whole data set (*n* = 2449) that were significantly associated with BMI, leptin, Glucose, C-peptide and/or IS_HOMA_. Metabolites are reported as significantly associated if in univariable analyses the multiplicity adjusted *p*-value (‘adj. *p*-value’) is ≤ 0.05 (using the method of Benjamini-Hochberg). Robustness analysis is performed on all measured metabolites, both annotated (*n* = 277) and non-annotated (*n* = 2172). NA: Not applicable (no metabolites significantly associated with the exposure). * metabolite selected by this method (adjusted *p*-value ≤ 0.1).

### Functional modules (MoDentify) and pathway analysis

We applied network analysis to identify sets of correlating metabolites that are coordinately associated with an exposure (BMI, leptin, glucose, C-peptide, IS_HOMA_).

**Fig. 2:**
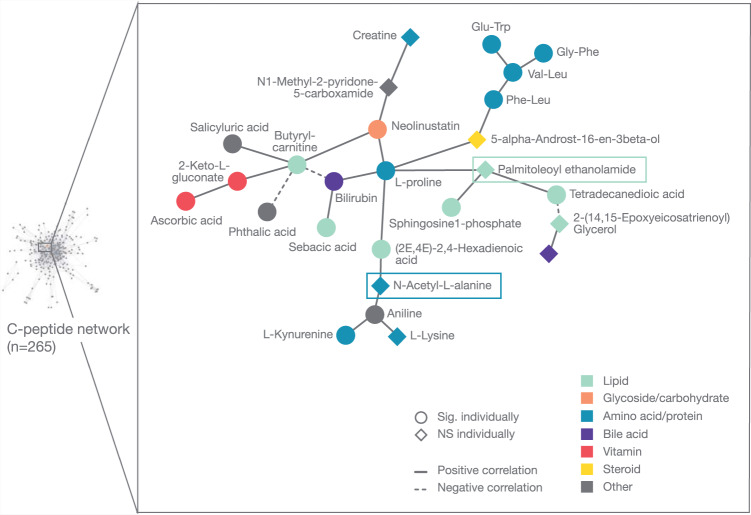
Subnetwork of metabolites (*n* = 26) that correlate with palmitoleoyl ethanolamide and are coordinately associated with C-peptide. The subnetwork is part of the network of all metabolites associated with C-peptide. The nodes represent metabolites and the edges significant partial correlations between metabolites after multiple testing correction (Benjamini-Hochberg, *p* ≤ 0.1). Round nodes correspond to metabolites significantly associated with C-peptide in the network but not when considered alone. Diamond nodes represent metabolites significantly associated with C-peptide also when considered alone. The model is adjusted for gestational age [days], maternal age [years] and processing time [minutes].

Because palmitoleoyl ethanolamide and N-acetyl-L-alanine were the most robust candidates, we sought to explore with which metabolites they correlate in the C-peptide network. Palmitoleoyl ethanolamide was part of two subnetworks and N-acetyl-L-alanine was part of three subnetworks (Supplementary Fig. 3). Because both metabolites shared a common subnetwork and this subnetwork was also the one including highest number of metabolites (*n* = 26), we selected it for graphical representation (Fig. 2) and pathway analysis (Supplementary Fig. 4).

Most metabolites included in this subnetwork were amino acids or proteins (*n* = 9, 35%), followed up by lipids (*n* = 7, 27%). Palmitoleoyl ethanolamide was directly correlated with L-proline, sphingosine-1-phosphate and tetradecanedioic acid concentration (Fig. 2). N-acetyl-L-alanine was directly correlated with (2E,4E)-2,4-hexadienoic acid and aniline (Fig. 2).

All the metabolites (*n* = 26) included in the subnetwork were selected for pathway analysis. However, no pathway was significantly enriched (Supplementary Fig. 4).

### The metabolome in healthy or unhealthy obesity

Obesity is metabolically heterogeneous and comprises individuals with normal (healthy) and dysregulated (unhealthy) metabolism [20]. We tested for potential differences in pre-selected metabolites (Supplementary Table 2) between women with overweight-obesity and low IS_HOMA_ (*n* = 9) and women with overweight-obesity, but normal IS_HOMA_ (*n* = 23) (Table 4). Two metabolites: tryptamine and 2,3,4,9-tetrahydro-1H-carboline-3-carboxylic acid were inversely associated with IS_HOMA_. The association remained significant in multivariable analysis after adjusting for confounders (Table 5).

**Table 4:** Characteristics of the sub-cohort (BMI ≥ 25) stratified by maternal IS_HOMA_.

	IS_HOMA_ ≥ 0.5 (*n* = 23)			IS_HOMA_ < 0.5 (*n* = 9)			
	*n*	median	IQR	*n*	median	IQR	*p*-value
Gestational age (days)	23	48	35–64	9	35	35–42	0.12
Maternal age (years)	23	31	27–39	9	35	28–38	0.97
BMI (kg/m^2^)	23	27.1	25.8–28.9	9	27.4	26.6–29.3	0.59
Leptin (ng/ml)	23	17.4	12.2–23.1	9	16.3	11.5–24.7	0.66
Glucose (mmol/l)	23	4.6	4.2–5.0	9	5.5	5.2–6.0	0.006
C-peptide (pmol/l)	23	370.9	321.6–457.5	9	599.2	523.4–759.3	<0.0001

*IS*_*HOMA*_ homeostatic model assessment of insulin sensitivity, *BMI* body mass index, *IQR* interquartile range.

Given are the *p*-values of the Kruskal–Wallis test comparing IS_HOMA_ groups.

**Table 5:** Metabolites significantly associated with IS_HOMA_ in the sub-cohort analysis of healthy and unhealthy obesity.

Metabolite	Exposure	Effect estimate	Confidence interval (95%)	effect size (%)	*p*-value
Tryptamine	IS_HOMA_	−0.84	−1.62 to −0.05	0.37	0.038
2,3,4,9-Tetrahydro-1H-carboline-3-carboxylic acid		−0.82	−1.59 to −0.06	0.38	0.036

The influence of the exposure IS_HOMA_ on the metabolites is modelled using linear regression terms. The model is adjusted, assuming linear relationships, for gestational age [days], maternal age [years] and processing time [minutes]. The effect estimates are given in relation to changes in the exposure: IS_HOMA_ < 0.5 – ≥ 0.5. The 95% confidence intervals are given for the effect estimates. Effect size (%) refers to the coefficient of determination (R2) and is the proportion of the variance in the outcome variable that is predictable from the influence variables (R2 lies between 0 and 100%). Analyses of variance of the regression models were performed and *p*-values of *F*-tests are reported.

## Discussion

In this study we identified circulating serum metabolites associated with maternal obesity in the first trimester of pregnancy. We searched for associations between BMI (measure of obesity), leptin (measure of adiposity), glucose, C-peptide and IS_HOMA_ (measures of glucose-insulin metabolism) and circulating serum metabolites. Applying different data analysis approaches we identified 15 serum metabolites significantly associated with at least one of the exposures (maternal BMI, leptin, glucose, C-peptide or IS_HOMA_).

Pregnancy is a physiological state that requires metabolic adaptations in the mother to accommodate to the nutrient and oxygen demands of the fetus. Early in gestation, maternal metabolism favours the storage of nutrients whereas in late gestation, it switches to a catabolic state to increase the availability of nutrients to the fetus [32]. These changes in the maternal metabolism are in part mediated by hormones secreted by the placenta [33]. Due to the metabolic flexibility required in pregnancy, which may be impaired under some circumstances [34], and the placental contribution to the maternal metabolic pool, the metabolic profile differs up to 44% between non-pregnant and pregnant women already in their first trimester of pregnancy [35]. As pregnancy progresses, composition and concentration of metabolites change [36]. This metabolic heterogeneity between pregnant and non-pregnant women as well as between pregnancy trimesters calls for analysing metabolic changes in women with obesity taking gestational age into account. Even though our study was limited to the first trimester of pregnancy, we included gestational age as a confounder in the multivariable and network analyses.

Our study is the first to analyse the maternal metabolome in the first trimester of pregnancy using untargeted metabolomics. This technique has the advantage to detect all metabolites that are present in the sample and, hence, comprehensively captures the metabolome landscape.

Outside pregnancy, amino acid concentration is often increased in the blood of women with obesity [37–41]. In particular, concentrations of branched-chained amino acids (BCAA) alanine, leucine and isoleucine have been associated with obesity and with development of insulin resistance [39]. Strikingly, in the first trimester of pregnancy, BCAA concentrations do not seem to associate with BMI [16, 42], which is in line with our findings. A potential explanation for this discrepancy is that BCAA are a source of nitrogen taken up by the placenta and transferred to the fetus [16]. The only amino acid robustly associated with an exposure in our study was N-acetyl-L-alanine that was significantly associated with C-peptide in the multivariable and robustness analysis. N-acetyl-L-alanine can result from either acetylation of alanine by specific N-acetyltransferases, or from proteolytic degradation of N-acetylated proteins. N-acetylation of proteins is highly conserved in eukaryotes, contributes to their protection and stability, and has been found to be related to obesity [43].

The second metabolite robustly associated with an exposure (C-peptide) was palmitoleoyl ethanolamide. It is an N-acylethanolamide endogenously synthesized from palmitoleic acid [44]. N-acylethanolamides, also known as endocannabinoid-like compounds, share the biosynthetic and degradation pathways of endocannabinoids, but lack affinity for the cannabinoid receptors CB1 and CB2 [45]. Instead, palmitoeloyl ethanolamide has affinity for the GPR119 receptor [46] mainly expressed in pancreatic β-cells and enterocytes. This might establish the link between palmitoleoyl ethanolamide and insulin sensitivity. Upon palmitoleoyl ethanolamide binding, GPR119 activation leads to an increase in intracellular cAMP levels entailing secretion of the incretin hormone Glucagon-like peptide 1 (GLP-1). GLP-1 acts at several levels: (1) in the gastrointestinal tract, GLP-1 inhibits gastric emptying and decreases appetite and (2) in the pancreas it stimulates insulin production and inhibits glucagon secretion [47]. Recently, GLP-1 receptor has been identified in term placenta, suggesting that the endocrine function of GLP-1 can also extend to this organ [48].

In an attempt to identify metabolites differing between ‘healthy’ and ‘unhealthy’ women with overweight-obesity, we classified women based on their IS_HOMA_ and compared the metabolites identified at the pre-selection step. We found tryptamine and 2,3,4,9-tetrahydro-1H-carboline-3-carboxylic acid (tryptophan by-products) to be inversely associated with insulin sensitivity. The sample size of the sub-cohort (*n* = 34) is small and we are underpowered to draw firm conclusions. However, the robust finding of associations between metabolic traits and tryptophan by-products suggests relevance of tryptophan metabolism in the regulation of the glucose-insulin axis and obesity [49, 50]. Similar to POEA, this association could be mediated by GLP-1 [49].

The fact that palmitoleic acid is the precursor of palmitoleoyl ethanolamide may suggest an influence of diet. However, in a previous study we did not find a significant association between C-peptide and palmitoleic acid in the same cohort [19]. As an alternative explanation, N-acetylethanolamides are endogenously synthesised and their concentration is regulated by synthesizing and degrading enzymes such as N-acyl phosphatidyl ethanolamine-specific phospholipase D (NAPE-PLD) and fatty acid amide hydrolase (FAAH) [51]. Thus, the observed increase in palmitoleoyl ethanolamide concentration in the serum of pregnant women with increasing C-peptide concentrations might be a consequence of altered enzymatic activity rather than differences in dietary patterns. Interestingly, the metabolites directly correlated with palmitoleoyl ethanolamide in the subnetwork analysis, e.g tetradecanedioic acid and D-erythro-sphingosine-1-phosphate, are also known to participate in glucose metabolism and insulin secretion [52, 53]. In a study with Sprague-Dawley rats fed with a high fat diet, parenteral administration of palmitoeloyl ethanolamide resulted in weight loss and improved insulin sensitivity [44]. This may establish a causal link between palmitoleoyl ethanolamide concentration and the parameters of the metabolic syndrome. Current knowledge on palmitoleoyl ethanolamide is sparse and more studies addressing the biochemistry and biology of this endocannabinoid-like lipid are needed.

Our study has several strengths. First, smokers were excluded not by self-report, but based on presence of cotinine in blood. Their exclusion avoids bias due to the known effect of smoking on basal metabolism [54] and pregnancy [55]. Second, in addition to BMI, defining obesity, and circulating maternal leptin as proxy for adiposity, we included maternal glucose, C-peptide and IS_HOMA_ as metabolic exposures since a proportion of individuals with obesity may be metabolically ‘healthy’ [20]. This approach proved better suited to understand fatty acid metabolism in the first trimester of pregnancy [19]. Third, we used untargeted metabolomics avoiding inherent selection bias of metabolites and offering the possibility to identify novel metabolites. Fourth, the use of stringent statistical methods (multivariable analysis adjusting for confounders and machine learning algorithms) ensured the identification of only very robust candidates. This is also corroborated by similar hits for e.g. N-acetyl-L-alanine and palmitoleoyl ethanolamide, in the robustness analysis, thus adding confidence to our results. Fifth, the combination with integrative analysis (network and pathway) allowed the identification of metabolite clusters changed in a coordinated way.

Some limitations also need to be mentioned. First, the small number of women with obesity in our cohort may limit representativeness of the results. To overcome this limitation, the overweight and obese groups were combined (30.6%) and BMI was included in the models as both categorical and continuous variable. Second, in our multivariable analysis, the exposure variables (BMI, leptin, glucose, C-peptide, IS_HOMA_) and confounders explained only between 5 and 30% of the metabolite variance. This is a common issue in -omics studies. When the association between maternal glucose during pregnancy and newborn size was analyzed, maternal fasting plasma glucose only explained 3–10% of the variation in newborn weight [12]. This low predictive ability reflects that metabolism is the result of many interacting exposures adding residual confounding. Dietary habits could be one confounder, although they have less influence on the metabolic milieu than pre-pregnancy BMI [16]. Further confounders are ethnicity, socio-economic disadvantage and stress levels, but we do not have this information available. Third, despite the advantage of untargeted metabolomics being a hypothesis-free method, signal annotation is oftentimes challenging. To counteract this limitation, our robustness analysis included all annotated and non-annotated metabolites to select the most prominent candidates. However, pathway analysis is sensitive to the lack of annotation. Non-annotated metabolites cannot be assigned to a pathway leading to misrepresentation of enriched pathways. We also do not know how pregnancies would have continued if allowed to progress, which precluded identification of biomarkers for adverse pregnancy outcomes. The metabolomics field is relatively new and still in development. In the future, advances in the annotation process will provide a better characterization of the whole metabolome.

This is the first study comparing the maternal metabolome in the first trimester of pregnancy considering proxies of obesity (BMI) and adiposity (leptin) as well as of the glucose-insulin axis (glucose, C-peptide, IS_HOMA_). We have identified several relevant metabolites and shown that N-acetyl-L-alanine and palmitoleoyl ethanolamide are robustly associated with C-peptide. This demonstrates that the serum metabolome of the pregnant woman is affected already very early in pregnancy by BMI and a disturbed glucose-insulin axis. Thus, attempts to normalize metabolism to prevent development of GDM and adverse pregnancy outcomes should begin very early in pregnancy or, ideally, before conception, but implementing this in the real world faces challenges.

## Supplementary information

Final_Submission_Provisional_Acceptance_Revised_Suppl-Material

## Data Availability

The datasets generated during and/or analysed during the current study are not publicly available due to privacy/ethical restrictions but are available from the corresponding author on reasonable request.
