# Supplementary material for: Distinct maternal metabolites are associated with obesity and glucose-insulin axis in the first trimester of pregnancy
Source: Int J Obes (Lond). 2023 Apr 7;47(7):529–37. doi: 10.1038/s41366-023-01295-4 (PMC10299907; doi:10.1038/s41366-023-01295-4)
Supplement: Supplementary file 1 — Final_Submission_Provisional_Acceptance_Revised_Suppl-Material [file 41366_2023_1295_MOESM1_ESM.docx]

**Revised Supplementary Information**

**Supplementary Information**

**Supplementary Materials and Methods**

1. Metabolomics analysis
2. Statistical Analysis
   1. Data pre-processing for extreme outliers
   2. Univariable analyses

2.3 Machine learning analysis

2.4 Network analysis

**Supplementary Tables and Figures**

**Supplementary Table 1**. Metabolites identified in the pre-selection step

**Supplementary Table 2**. Sub-group analysis of the metabolome (19 metabolites) of women with overweight/obesity with normal (IS_HOMA_ ≥ 0.5) or low (IS_HOMA_ < 0.5) insulin sensitivity.

**Supplementary Figure 1.** Associations modelled with restricted cubic spline terms.

**Supplementary Figure 2**. Robustness analysis.

**Supplementary Figure 3.** Subnetworks, within the C-peptide network that include N-acetyl-L-alanine or palmitoleoyl ethanolamide.

**Supplementary Figure 4.** Pathway analysis of the subnetwork.

**Supplementary Materials and Methods**

# **Metabolomics analysis**

Analytes were separated on a Zorbax Eclipse Plus C18 guard (2.1 × 50 mm and 1.8 μm particle size, Agilent Technologies, Santa Clara, CA, USA) and an analytical column (2.1 × 150 mm and 1.8 μm particle size, Agilent Technologies, Santa Clara, CA, USA) kept at 40°C. The analytes were eluted using a flow rate of 400 μL/min with the following composition of eluent A (0.1% formic acid) and eluent B (0.1% formic acid, acetonitrile) solvents: 3% B from 0 to 1.5 min, 3–40% B from 1.5 to 4.5 min, 40‐95% B from 4.5 to 7.5 min, 95 % B from 7.5 to 10.1 min and 95 to 3% B from 10.1 to 10.5 min before equilibration for 3.5 min with the initial conditions. The flow from the HPLC was coupled to a Q Exactive HF mass spectrometer (Thermo Fisher Scientific, Bremen, Germany) for mass spectrometric analysis in both positive and negative ion mode using the following general settings for MS1 mode: resolution: 120.000, AGC target: 3e6, maximum injection time: 200 ms, scan range 65-975 m/z and lock mass: 391.28429/112.98563 (pos/neg mode). For compound fragmentation MS1/ddMS2 mode were used with the following general settings: resolution: 60.000/15.000, AGC target: 1e6/1e5, maximum injection time: 50/100 ms, scan range 65-975 m/z, loop count: 5, isolation with: 2 m/z and normalized collision energy: 35/38 (pos/neg). A pooled sample was generated and used for quality control (QC) and compound fragmentation. The sequence was constructed with analysis of the blanks first followed by system equilibrations runs using QC sample, MS1/ddMS2 runs for compound fragmentation on QC sample, another QC run and finally, analysis of the samples in randomized order in MS1 mode with a QC sample injected every ninth run. A MS2 inclusion list was generated from the blanks and the last equilibration runs using Compound Discover v. 3.0 (Thermo Fisher Scientific) to ensure fragmentation of the later extracted features. The features found in the blank were removed from the inclusion list if they were not > 5 x more abundant in the QC samples.

Raw data was processed with MzMine (v 2.42) ^1^. In brief, the following modules were used: Mass detection, ADAP chromatogram builder, ADAP deconvolution, Join aligner, Isotopic peak grouper, Gap filling (same RT and m/z range) and Identification in local spectra database search; all with 5 ppm mass tolerance and 0.25 RT tolerance when possible. Final peaklist included features found in at least 75% of the samples, which had at least 2 peaks in an isotope pattern. Compounds were annotated at Metabolomics Standards Initiative (MSI) ^2^ level 2 using local MS/MS spectra databases of National Institute of Standards and Technology 17 (NIST17) and MassBank of North America (MoNA). MSI level 4 annotations were achieved by searching the molecular formula in Human Metabolite Databse ^3^. After compound annotation, the datasets were corrected for signal drift using statTarget ^4^. Finally, the signal was signal normalized using the QC sample ^5^.

# **2. Statistical Analysis**

## **2.1 Data pre-processing for extreme outliers**

Before log2-transformation of metabolite values for further analyses, extreme outliers were replaced. Extreme outliers exceeding the range [Mean ± 4 * SD] were interpreted as measurement errors or contamination and counted as missing (Mean – mean of metabolite values, SD - standard deviation of metabolite values). Missings denoting extreme outliers were imputed simultaneously by the k-nearest neighbours approach (k=10) ^6^.

## **2.2 Univariable analyses**

The association between metabolite abundance (continuous variable) and a specific exposure variable was analyzed by either Kruskal-Wallis tests (if exposure variable categorized) or by tests for association using Spearman’s correlation coefficient (if exposure variable continuous). Additionally, univariable linear regression models were used to assess the effect of the categorized and continuous exposures on the metabolites as outcomes. ANOVA analysis was performed using F-tests. P-values were adjusted for multiple testing for each test by the method of Benjamini-Hochberg ^7^, which allows controlling the false discovery rate (FDR).

## **2.3 Machine learning analysis**

The total set of annotated metabolites was analyzed for its ability to classify the binarized exposures (categorized by their median) using the R-package “biosigner” ^8^. The package implements a wrapper for the binary classifiers partial least squares discriminant analysis (PLS-DA), random forest (RF) and support vector machine (SVM). The algorithm is based on training and test subsets and selects repeatedly a metabolite signature for each classifier until the selected signature is stable, i.e. the set of metabolites does not change in the next iteration step. The algorithm returns the tier of each metabolite for the classifiers: tier S corresponds to the final signature, i.e., metabolites, which have been found significant in all the selection steps; metabolites with tier A have been found significant in all, but the last selection. We consider a metabolite as selected by “biosigner” if it is in tier A or S for at least one of the three methods (PLS-DA, RF, SVM).

## **2.4 Network analysis**

Phenotype-driven metabolomics network analysis was performed as an additional statistical approach to screen for promising metabolites. The method of Do et al., implemented in R-package “MoDentify”^9^, was used for exposure-driven module identification in the metabolomics network.

For network inference, MoDentify estimates Gaussian graphical models (GGMs) to reconstruct metabolic pathways from metabolomics data ^4^. Nodes represent metabolites and edges represent significant partial correlation between two nodes after multiple testing correction (Benjamini-Hochberg at 10% significance level). Partial correlations represent associations between two variables corrected for all remaining variables in multivariable Gaussian distributions. We included the covariates gestational age, maternal age, and processing time of blood in the model. To identify functional modules, MoDentify uses a score maximization approach, which accounts for the considered exposure and the covariates. To calculate the score of a candidate module, the module has to be summarized in the module representative value, which aggregates the total abundance of metabolites from the pathway into a single value. Here, we used the average approach, where the module representative value is calculated as the average of all z-scored metabolite concentrations in the module. The significance of the modules is assessed by correcting for the total number of nodes in the underlying network (Benjamini-Hochberg at 5% significance level).

# **Supplementary Table 1.** Metabolites identified in the pre-selection step.

| **Metabolite** | **Exposures** | **Continuous** | | | **Categorized** | | **Feature Selection** |
| --- | --- | --- | --- | --- | --- | --- | --- |
|  |  | **non-parametric (adj, *p*-value)** | | **linear regression (adj. *p*-value)** | **non-parametric (adj. *p*-value)** | **linear regression (adj. *p*-value)** | **Machine Learning** |
|  |  |  |  |  |  |  |  |
| Stachydrine | BMI | * |  | |  |  |  |
| Phenylalanine-Threonine |  | * |  | |  |  |  |
| Serine-Tyrosine |  | * |  | |  |  |  |
| Proline-Hydroxyproline |  | * |  | |  |  |  |
| S-Methyl-L-cysteine |  | * |  | |  |  |  |
| Phenylalanine-Valine | Leptin |  |  | |  | * |  |
| Tryptamine | Glucose |  |  | | * | * |  |
| 2,3,4,9-Tetrahydro-1H-carboline-3-carboxylic acid |  |  |  | | * | * |  |
| Hexamethylphosphoramide |  |  |  | | * | ‡ |  |
| Phenylalanine-Threonine |  |  |  | | * |  |  |
| Serine-Tyrosine |  |  |  | | ‡ |  |  |
| 5-(Tetradecyloxy)-2-furoic acid |  |  |  | | * | * |  |
| 15S-Hydroperoxy-11Z,13E-eicosadienoic acid |  | † | * | | † | † |  |
| 1-Myristoyl-sn-glycero-3-phosphocholine |  |  |  | | * | * |  |
| 3,5-dihydroxyphenyl dodecyl benzene-1,3-diol |  | ‡ |  | | ‡ | ‡ |  |
| Uridine | C-peptide |  |  | |  | * | § |
| N-Acetyl-L-alanine |  |  |  | |  | ‡ |  |
| 7α -(…)-diol glucuronide |  |  |  | |  | * |  |
| Palmitoleoyl ethanolamide |  |  |  | |  | ‡ |  |
| Androsterone glucuronide |  |  |  | |  |  | § |
| Stachydrine |  |  |  | |  |  | § |
| Diketene |  |  |  | |  |  | § |
|  |  |  |  | |  |  |  |
| 1-Myristoyl-sn-glycero-3-phosphocholine | IS_HOMA_ |  | ‡ | |  | ‡ |  |
| S-Methyl-L-cysteine |  |  |  | |  |  | § |

To select the most prominent candidates at the pre-selection step, two statistical approaches were used: 1) univariable analysis and 2) feature selection. In the univariable analysis, the exposures were analyzed as a) continuous and b) categorized in two groups based on above/below the median value. The univariable association between metabolite abundance and a specific exposure was analyzed by either Kruskal-Wallis tests (for categorized variables) or tests for association based on Spearman’s correlation coefficient (for continuous variables). The *p*-value was adjusted by the method of Benjamini-Hochberg (FDR ≤ 0.1). For feature selection (combination of support vector machine, partial least squares discriminant analysis and random forest) the exposures were categorized based on above/below the median value. Pre-selected metabolic candidates are further explored in multivariable analysis adjusting for confounders (see Table 2).BMI: body mass index; IS_HOMA_: homeostatic model assessment of insulin sensitivity; 7α -(…)-diol glucuronide: 7α,17α-Dimethyl-5β-androstane-3α,17β-diol glucuronide; * Adjusted *p*-value ≤ 0.1; ‡ Adjusted *p*-value ≤ 0.05; † Adjusted *p*-value ≤ 0.01; § Metabolites which have been found significant in all the selection steps or in all but the last selection step using the R package “biosigner”.

# **Supplementary Table 2**. Sub-group analysis of the metabolome (19 metabolites) of women with overweight/obesity with normal (IS_HOMA_ ≥ 0.5) or low (IS_HOMA_ < 0.5) insulin sensitivity.

| **Metabolite** |  | |
| --- | --- | --- |
|  | **non-parametric (adj. *p*-value)** | **linear regression (adj. *p*-value)** |
|  |  |  |
| Stachydrine |  |  |
| Phenylalanine-Threonine |  |  |
| Serine-Tyrosine |  |  |
| Proline-Hydroxyproline |  |  |
| S-Methyl-L-cysteine |  |  |
| Phenylalanine-Valine |  |  |
| Tryptamine | † | † |
| 2,3,4,9-Tetrahydro-1H-carboline-3-carboxylic acid | † | † |
| Hexamethylphosphoramide |  |  |
| 5-(Tetradecyloxy)-2-furoic acid |  |  |
| 15S-Hydroperoxy-11Z,13E-eicosadienoic acid |  |  |
| 1-Myristoyl-sn-glycero-3-phosphocholine |  |  |
| 3,5-dihydroxyphenyl dodecyl benzene-1,3-diol |  |  |
| Uridine |  |  |
| N-Acetyl-L-alanine |  |  |
| 7α -(…)-diol glucuronide |  |  |
| Palmitoleoyl ethanolamide |  |  |
| Androsterone glucuronide |  |  |
| Diketene |  |  |

To investigate the metabolome of women with overweight/obesity, but with normal insulin sensitivity, the metabolites identified in the pre-selection step (n = 19; Supplementary Table1) were compared between the low and normal insulin sensitivity group. The IS_HOMA_ threshold to classify the women into normal or low insulin sensitive women is based on the lower quartile of IS_HOMA_ in the total cohort (lower quartile of IS_HOMA_: 0.5; normal IS_HOMA_ ≥ 0.5, n = 23; low IS_HOMA_ < 0.5; n = 9). Associations between metabolites and IS_HOMA_ were tested by Kruskal-Wallis test and linear regression analyses (adjusting for multiple testing by the method of Benjamini Hochberg, FDR ≤ 0.1). IS_HOMA_: homeostatic model assessment of insulin sensitivity. † Adjusted *p*-value ≤ 0.01.

# **Supplementary Figure 1**. Associations modelled with restricted cubic spline terms.


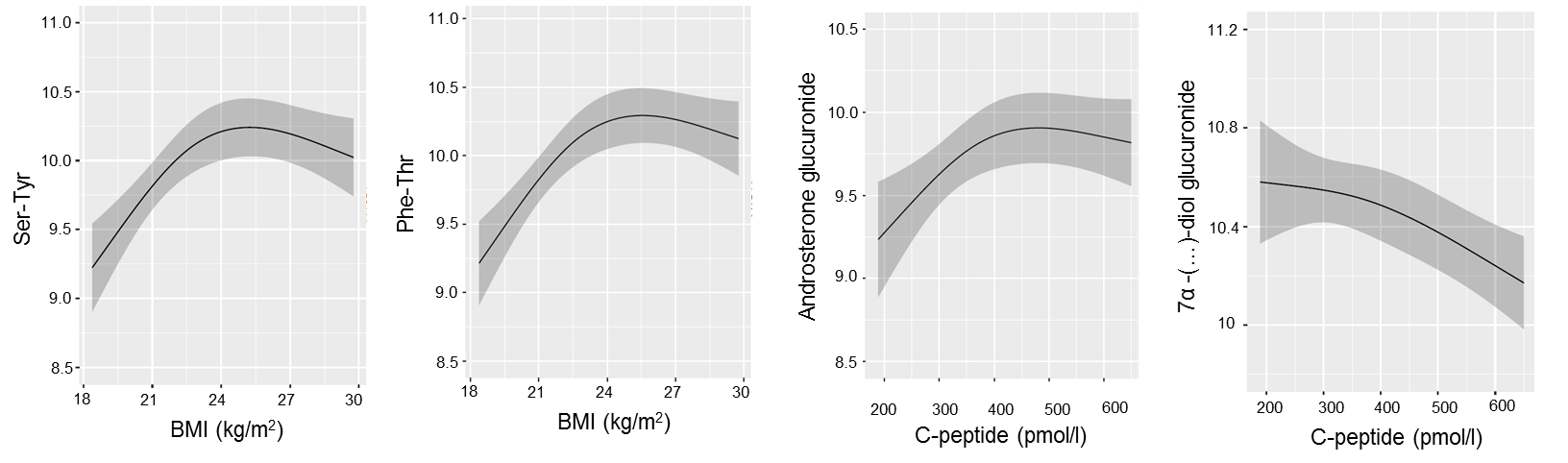


The influence of selected exposures on the metabolites which are modelled using restricted cubic spline terms. The model is adjusted, assuming linear relationships, for gestational age (days), maternal age (years) and processing time (minutes) and includes an interaction term between the exposure and gestational age. BMI: body mass index; 7α -(…)-diol glucuronide: 7α,17α-Dimethyl-5β-androstane-3α,17β-diol glucuronide.

# **Supplementary Figure 2**. Robustness analysis. Number of metabolites (annotated and non-annotated) significantly associated with one of the exposures.


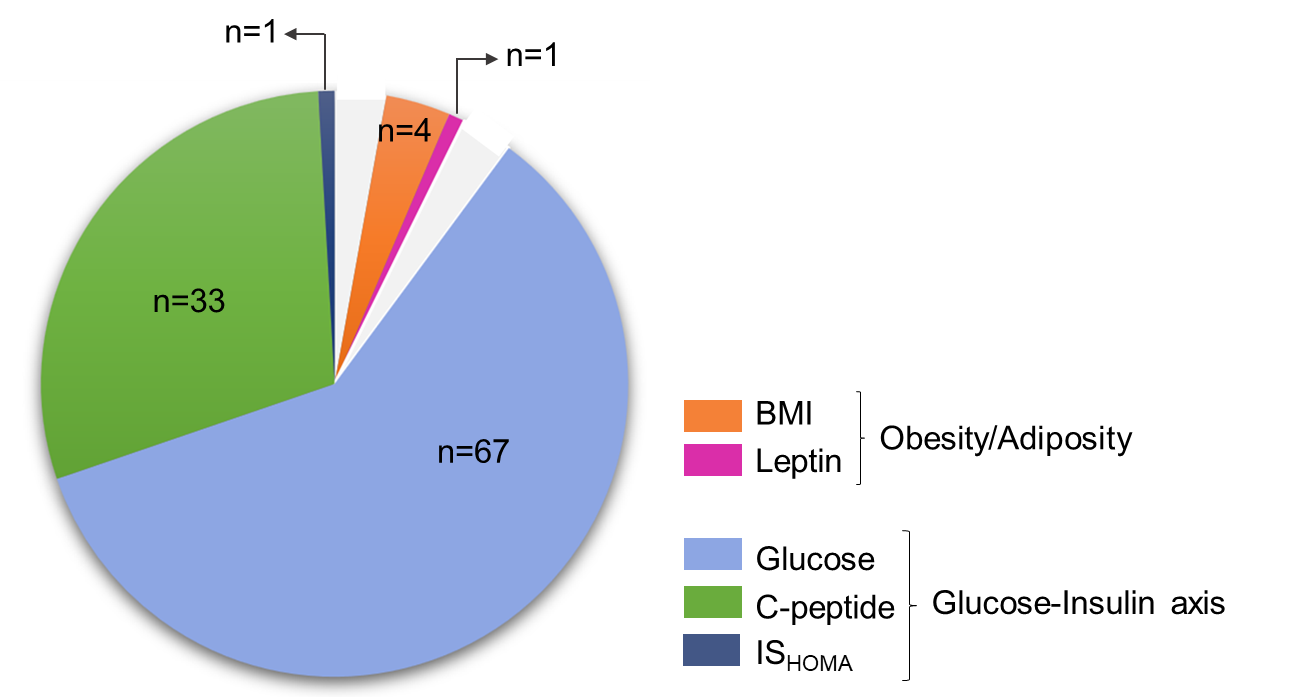
One-hundred and six different metabolites (4.3% of the total number of metabolites) were associated with at least one of the exposures (number of metabolites: BMI = 4, leptin = 1, Glucose = 67, C-peptide = 33, IS_HOMA_ = 1).

#
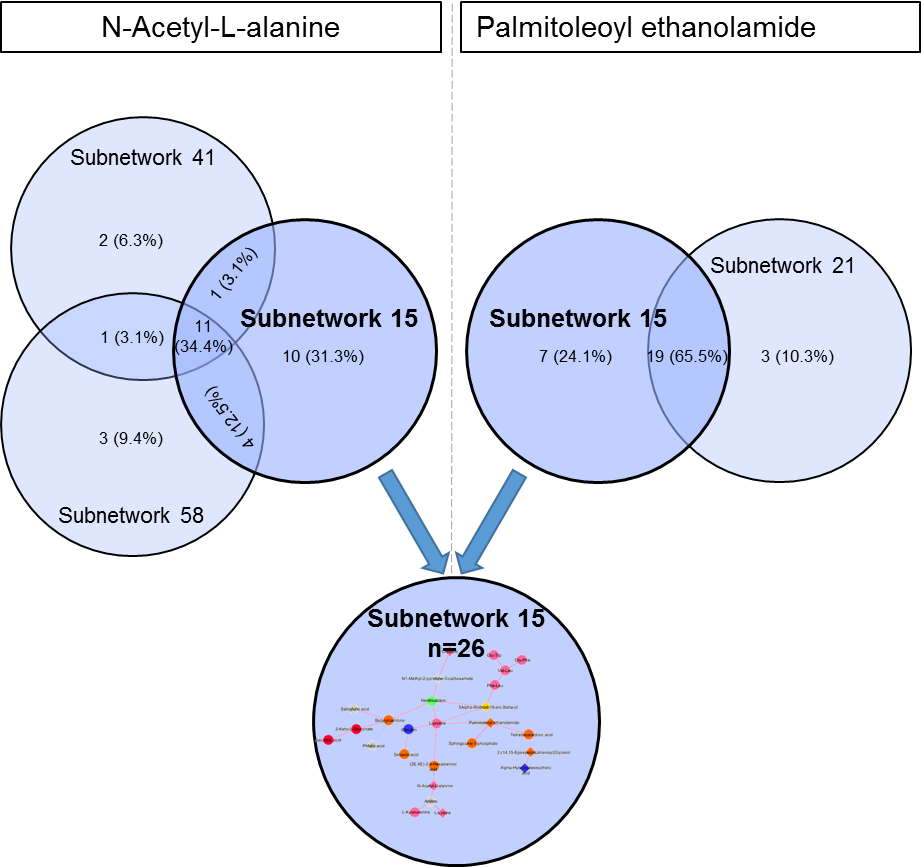
**Supplementary Figure 3.** Subnetworks, within the C-peptide network that include N-acetyl-L-alanine or palmitoleoyl ethanolamide.

Both metabolites (N-acetyl-L-alanine and palmitoleoyl ethanolamide) are part of subnetwork 15. This subnetwork was used for graphical representation and pathway enrichment. The number and percentage of metabolites in each subnetwork is indicated. The percentage was calculated as: n° of metabolites in the subnetwork / n° of different metabolites in all the subnetworks that include the metabolite of interest * 100.

#
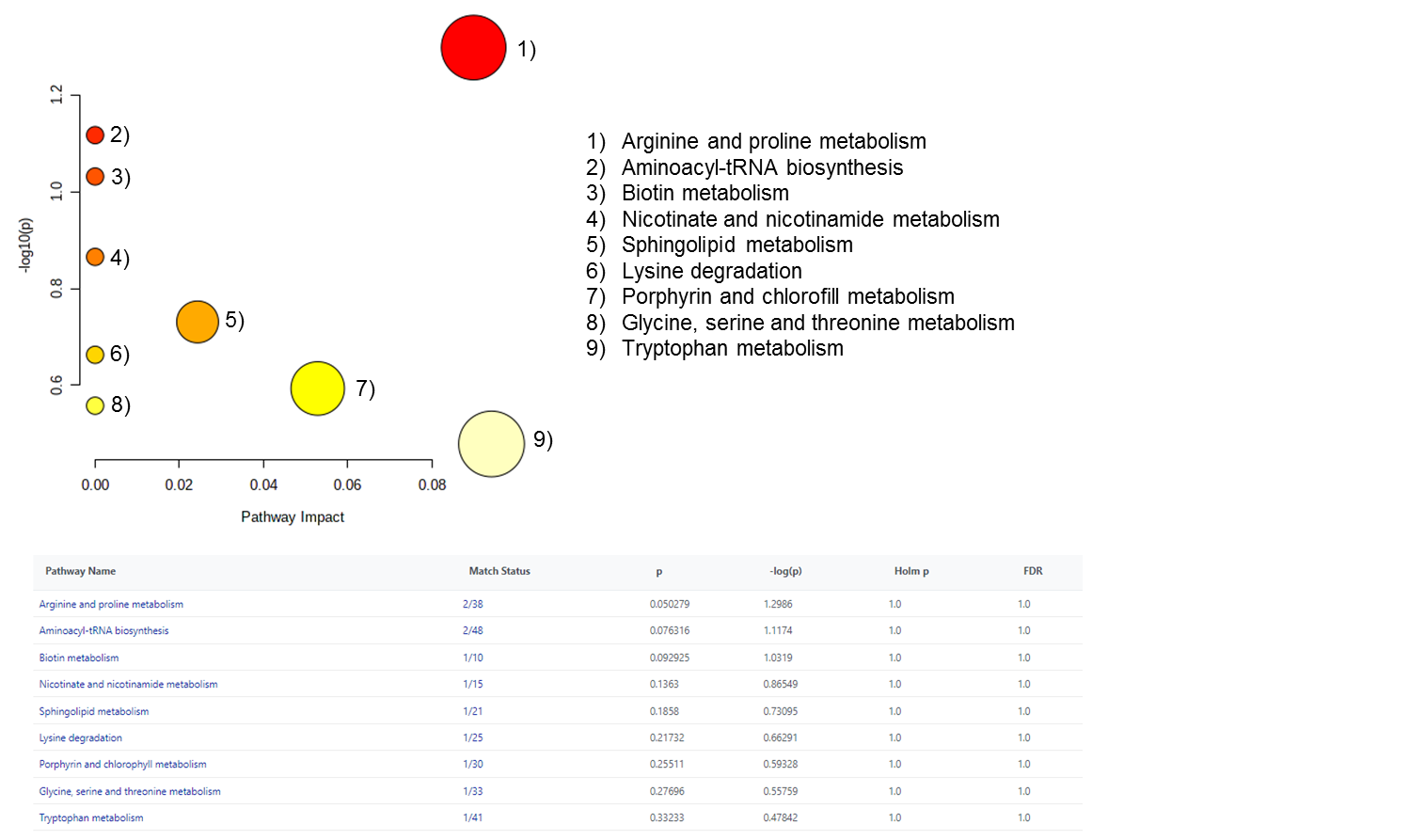
**Supplementary Figure 4.** Pathway analysis of the subnetwork of metabolites (n=26) that correlate with N-acetyl-L-alanine and palmitoleoyl ethanolamide and are coordinately associated with C-peptide.

| **Pathway name** | **Match status** | ***p*-value** | ***p*-value (Holm adj.)** | **FDR** |
| --- | --- | --- | --- | --- |
| Arginine and proline metabolism | 2/38 | 0.05 | 1.0 | 1.0 |
| Aminoacyl-tRNA biosynthesis | 2/48 | 0.08 | 1.0 | 1.0 |
| Biotin metabolism | 1/10 | 0.09 | 1.0 | 1.0 |
| Nicotinate and nicotinamide metabolism | 1/15 | 0.14 | 1.0 | 1.0 |
| Sphingolipid metabolism | 1/21 | 0.19 | 1.0 | 1.0 |
| Lysine degradation | 1/25 | 0.22 | 1.0 | 1.0 |
| Porphyrin and chlorophyll metabolism | 1/30 | 0.26 | 1.0 | 1.0 |
| Glycine, serine and threonine metabolism | 1/33 | 0.28 | 1.0 | 1.0 |
| Tryptophan metabolism | 1/41 | 0.33 | 1.0 | 1.0 |

All metabolites correlating with N-acetyl-L-alanine and palmitoleoyl ethanolamide and functionally regulated by C-peptide (Figure 2) were selected for pathway analysis in MetaboAnalyst. Pathway enrichment was assessed with a hypergeometric test and the KEGG (*Homo sapiens*) library was used as reference. Match Status: number of metabolites in the dataset that are included in the KEGG pathway/Total number of metabolites comprised in the KEGG pathway; p-value (Holm adj.): p-value after Holm adjustment; FDR: false discovery rate.

# **Bibliography**

1. Pluskal T, Castillo S, Villar-Briones A, Oresic M. MZmine 2: modular framework for processing, visualizing, and analyzing mass spectrometry-based molecular profile data. *BMC Bioinformatics* 2010; **11:** 395.

2. Salek RM, Steinbeck C, Viant MR, Goodacre R, Dunn WB. The role of reporting standards for metabolite annotation and identification in metabolomic studies. *Gigascience* 2013; **2**(1)**:** 13.

3. Wishart DS, Feunang YD, Marcu A, Guo AC, Liang K, Vazquez-Fresno R *et al.* HMDB 4.0: the human metabolome database for 2018. *Nucleic Acids Res* 2018; **46**(D1)**:** D608-D617.

4. Luan H, Ji F, Chen Y, Cai Z. statTarget: A streamlined tool for signal drift correction and interpretations of quantitative mass spectrometry-based omics data. *Anal Chim Acta* 2018; **1036:** 66-72.

5. Chong J, Wishart DS, Xia J. Using MetaboAnalyst 4.0 for Comprehensive and Integrative Metabolomics Data Analysis. *Curr Protoc Bioinformatics* 2019; **68**(1)**:** e86.

6. Do KT, Wahl S, Raffler J, Molnos S, Laimighofer M, Adamski J *et al.* Characterization of missing values in untargeted MS-based metabolomics data and evaluation of missing data handling strategies. *Metabolomics* 2018; **14**(10)**:** 128.

7. Benjamini Y, Hochberg Y. Controlling the False Discovery Rate: A Practical and Powerful Approach to Multiple Testing. *Journal Royal Statistical Society B* 1995; **57**(1)**:** 289-300.

8. Rinaudo P, Boudah S, Junot C, Thevenot EA. biosigner: A New Method for the Discovery of Significant Molecular Signatures from Omics Data. *Front Mol Biosci* 2016; **3:** 26.

9. Do KT, Rasp DJN, Kastenmuller G, Suhre K, Krumsiek J. MoDentify: phenotype-driven module identification in metabolomics networks at different resolutions. *Bioinformatics* 2019; **35**(3)**:** 532-534.
